# Altered miR-223 Expression in Sputum for Diagnosis of Non-Small Cell Lung Cancer

**Published:** 2017

**Authors:** Abouzar Bagheri, Hamid Reza Khorram Khorshid, Seyed Javad Mowla, Hassan Ali Mohebbi, Azam Mohammadian, Mehdi Yaseri, Masoud Solaymani-Dodaran, Masih Sherafatian, Mahmood Tavallaie

**Affiliations:** 1. Genetic Research Center, University of Social Welfare and Rehabilitation Sciences, Tehran, Iran; 2. Department of Molecular Genetics, Faculty of Biological Sciences, Tarbiat Modares University, Tehran, Iran; 3. Trauma Research Center, Baghiyatallah University of Medical Sciences, Tehran, Iran; 4. Department of Chemistry, Faculty of Science, Ferdowsi University of Mashhad, Mashhad, Iran; 5. Department of Epidemiology and Biostatistics, Tehran University of Medical Sciences, Tehran, Iran; 6. Iran University of Medical Sciences, Tehran, Iran; 7. Human Genetic Research Center, Baqiyatallah University of Medical Sciences, Tehran, Iran

**Keywords:** MicroRNAs, Non-small cell lung carcinoma, Sputum

## Abstract

**Background:**

Diagnosis of Non-small Cell Lung Cancer (NSCLC) at an early stage is a daunting challenge due to the deficiency of specific noninvasive markers. MicroRNAs (miRNAs) play important roles in the initiation and progression of NSCLC. Measuring miRNA expression levels could provide a potential approach for the diagnosis of NSCLC. Our goals were to examine miR-223, miR-212, miR-192, miR-3074, SNORD33 and SNORD37 expression levels in tissue and sputum of NSCLC patients and cancer free subjects for molecular diagnosis of NSCLC.

**Methods:**

Relative expressions of miR-223, miR-212, miR-192, miR-3074, SNORD33 and SNORD37 were examined with quantitative real-time RT-PCR assay in tissue and sputum obtained from 17 NSCLC patients and 17 controls.

**Results:**

miR-3074 was upregulated in tissue samples of NSCLC patients compared with control group. miR-223 was upregulated, miR-212 and SNORD37 were downergulated in sputum samples of patients compared with controls. miR-223 quantification produced 82% sensitivity and 95% specificity with areas under the ROC curve at 0.90 in detection of NSCLC.

**Conclusion:**

miR-223 clearly discriminated cancer patients from cancer-free subjects and our results suggest that miR-223 could be a diagnostic useful biomarker. The measurement of altered miRNA expression in sputum samples manifested the potential noninvasive approach for detection of lung cancer.

## Introduction

Lung cancer is the most common cause of cancer death worldwide ^1^. Unfortunately, initial symptoms of lung problems appear when the disease is in an advanced stage ^2^. Non-small Cell Lung Cancers (NSCLC) account for ∼80–85% of all lung cancer cases ^3^. More than 75% of NSCLCs are diagnosed when the disease is locally advanced or metastatic. This fact represents a current 5-year survival of less than 15% ^4,5^. Therefore, finding NSCLC in early stages is a realistic approach to reduce the mortality associated with NSCLC. While computed tomography seems hopeful in detection of NSCLC at a smaller size compared to a chest X-ray, the improved sensitivity is related to an increased false-positive rate. The fluorescence bronchoscopy exceeds at diagnosing centrally-located lung tumors. However, it is an invasive technique ^6,7^. The development of highly valid and noninvasive diagnostic procedure would simplify the early detection of NSCLC, which is clinically meaningful.

Small non-coding RNAs (sncRNAs) mainly consist of microRNAs (miRNAs) and small nucleolar RNAs (snoRNAs) ^8^. miRNAs can post-transcriptionally regulate the expression of myriad of different target genes including more than 30% of protein coding genes ^9^, thereby managing an extensive spread of biological functions such as cellular proliferation ^10^, apoptosis ^11^ and differentiation ^12^. Scientific emerging evidences suggest the potential involvement of altered miRNA expressions in the pathogenesis of human cancers ^13–15^. miRNAs may function as tumor suppressors or oncogenes, thus dysregulated expressions participate in cancer development and progression ^16,17^. Consequently, miRNAs can potentially be useful in the detection, classification, prognosis, and therapy of human malignancies ^18^.

Recently, new and unexpected functions of other types of small ncRNAs have been discovered and investigators found that snoRNA expression in cancers is as variable as miRNA expression ^19^. Some snoRNAs could be processed to produce molecules like miRNA which drive post-translational gene silencing on complementary mRNAs ^20–22^. Expression of snoRNAs could be detected in biological fluids, making them potentially applicable biomarkers ^23^.

Sputum is the most fluently accessible specimen that includes the pathogenically relative cell types; furthermore, collecting sputum is noninvasive, fast, and economical, which are prominent features to be an ideal sample type for population screening. These characteristics cause molecular analysis of sputum to be an important target for the investigation of lung cancer biomarkers ^24^.

It is hypothesized that simultaneous assessment of a panel of ncRNAs could provide a highly sensitive and specific diagnostic test for detection of NSCLC. To verify the hypothesis, a panel of significant ncRNAs, including miR-223, miR-212, miR-192, miR-3074, SNORD33 and SNORD37 was selected to analyze tissue and sputum of NSCLC patients.

## Materials and Methods

To determine the clinical significance of dysregulated expressions of ncRNAs in tissue and sputum for diagnosis of NSCLC, expression changes of 6 cancer-associated ncRNAs, miR-223, miR-212, miR-192, miR-3074, SNORD33 and SNORD37, in 17 NSCLC patients and 17 cancer free subjects were evaluated.

### Sample collection

Lung tissue and sputum samples were collected from patients at Masih Daneshvari and Baghiat Allah Hospitals (Tehran, Iran). The research has been performed in accordance with the Declaration of Helsinki and has been approved by Ethics Committee of the University of Social Welfare and Rehabilitation Sciences, Tehran, Iran. All the participants agreed to the research plan and signed the written consent form and ethics permission was obtained for the research on samples.

Subjects in this study, 17 NSCLC patients 51–73 years old, had histopathologically confirmed primary NSCLC, stages I–IV, and medical history information and 17 cancer-free controls were sex, and age matched to the patients group (Table 1).

**Table 1.** Characteristics of 17 NSCLC patients and 17 cancer-free controls

	**Sex**		**Age (year)**	**Histologic types**		**Stage**			

	**Male**	**Female**	**-**	**SCC**	**AC**	**I**	**II**	**III**	**IV**
**Cancer patients**	15	2	51–73	6	11	2	3	5	7
**Cancer-free controls**	15	2	48–71	--		--			

Lung tissue specimens were immediately immersed in RNAlater buffer (Applied Biosystems, USA) and stored at −80*°C* for RNA extraction. Prior to the collection of a sputum sample, patients rinsed their mouths with water, breathed deeply, held their breath and coughed. All expectorated sputum were collected into a sterile plastic sample container that was then sealed and stored at −80*°C* until further processing. Routine sputum cytology was not performed on the collected sputum samples because previous studies have shown that sputum cytology has a high rate of both false positives and false negatives ^25,26^.

### RNA isolation

1 *ml* of TRIzol (Ambion, USA) and 750 *μl* of TRIzol-LS (Ambion, USA) were added to the individual homogenized tissue and sputum samples, respectively. Samples were then reacted at room temperature for 5 *min*. Chloroform was added to extract RNA then 500 *μl* of isopropanol was added to precipitate RNA, which was then washed with 75% EtOH. RNA was then dissolved in nuclease-free water. The concentration and purity of the isolated RNA were determined by a NanoDrop and the integrity of the RNA was verified using RNase-free agarose gel electrophoresis.

### cDNA synthesis and real-time RT–PCR

Poly (A) tailing of RNA was performed by *Escherichia coli (E. coli)* poly (A) polymerase kit (New England Biolabs, UK), then reverse transcription reaction was carried out by anchored oligo (dT) primer (Table 2) and a reverse transcriptase kit (Thermo Scientific, USA). cDNA synthesis parameters were as follows: 42*°C* for 60 *min* and 70*°C* for 10 *min*. Quantitative real-time RT–PCR was performed using EvaGreen master mix (Solis BioDyne, Estonia) and specific primers for miR-223, miR-212, miR-192, miR-3074, SNORD33 and SNORD37 (Table 2). The PCR parameters were as follows: initial denaturation (one cycle at 95*°C* for 15 *min*); 40 cycles of denaturation, amplification, and quantification (95*°C* for 15 *s*, 58–64*°C* for 30 *s*, and 72*°C* for 5 *s*); and the melting curve (starting at 65*°C* and gradually increasing to 95*°C*). The miRNA expression was normalized to the levels of U6, and expression differences were calculated according to the standard curve and efficiency established for each primer set.

**Table 2.** Primer sequences used in real-time RT-PCR analysis

**Name**	**Forward**	**Reverse**
**miR-192**	GTGAGCTGACCTATGAATTGACA	GCGAGCACAGAATTAATACGAC
**miR-3074**	ACCATTCCTGCTGAACTGAG	GCGAGCACAGAATTAATACGAC
**SNORD37**	CACGATGTCTACTGAAGAAAGCCTG	GCGAGCACAGAATTAATACGAC
**SNORD33**	TTTCCCGACCATGAGATGAC	GCGAGCACAGAATTAATACGAC
**U6**	TTTCGCAAGGATGACACGC	GCGAGCACAGAATTAATACGAC
**miR-223**	(Pg4487-03, Parsgenome, Iran)	
**miR-212**	(Pg4487-03, Parsgenome, Iran)	
**Anchored Oligo (dT) Primer**	GCGAGCACAGAATTAATACGACTCACTATAGG (32bp) (T)12VN ^*^	

* V= G, A, C; N= G, A, T, C.

### Statistical analysis

All statistical analysis performed by R (R Core Team (2014), R: A language and environment for statistical computing. R Foundation for Statistical Computing, Vienna, Austria, URL http://www.R-project.org). P-value of <0.05 was considered statistically significant. Kolomogrov Smirnov test as well as Q-Q plot were applied to check the normal distribution of data. To compare the ncRNAs between cancer patients and controls, Mann-Whitney test was used. Also, to test the difference adjusted for the effect of age, sex, smoking and clinicopathologic characteristics, logistic regression was used. Furthermore, Receiver-Operator Characteristic (ROC) curve analysis was undertaken for each gene in the sputum specimens from cancer patients and cancer-free controls. Also, the Areas Under the ROC Curve (AUROCs) were calculated and the optimal threshold was chosen based on Youden’s J statistics, then sensitivity and specificity as well as diagnostic accuracy levels to distinguish control individuals from cancer patients, and corresponding thresholds were calculated for each ncRNA. To obtain the best combination of ncRNAs that can distinguish the cancer patients from controls, logistic regression was used.

## Results

### ncRNAs as biomarkers in sputum samples of NSCLC patients

miR-223 significantly increased in sputum of cancer patients compared to non-cancers (p<0.05). miR-223 overexpression resulted in 82% (95% CI, 0.63–1.00) sensitivity and 95% (95% CI, 0.86–1.00) specificity in the diagnosis of NSCLC. miR-212 significantly decreased in sputum of patients compared to controls (p<0.05). miR-212 underexpression resulted in 68% (95% CI, 0.46–0.90) sensitivity and 64% (95% CI, 0.41–0.87) specificity in the diagnosis of NSCLC. SNORD37 significantly decreased in sputum samples of NSCLCs compared to controls and resulted in 93.3% (95% CI, 0.46–0.90) sensitivity and 63.3% (95% CI, 0.41–0.87) specificity in the diagnosis of NSCLC (p<0.05). miR-192, miR-3074 and SNORD33 did not alter in sputum of patients compared to controls (p> 0.05), (Table 3). Prevalence of miR-223, miR-212, miR-192, miR-3074, SNORD33 and SNORD37 expressions detected in sputum was not associated with patient age, gender, histological tumor type and stage (p>0.05).

**Table 3.** Evaluated ncRNAs in NSCLC patients and cancer-free controls in sputum samples

**ncRNA**	p-value^*^	**Fold change (cancer/control)**	**AUC (95% CI)**	**Cutoffs**	**Sensitivity**	**Specificity**
**miR-223**	<0.05	19.87	0.90 (0.81–0.99)	4.83	82%	95%
**miR-212**	<0.05	0.21	0.69 (0.53–0.85)	0.65	45%	91%
**miR-3074**	>0.05	0.89	0.65 (0.51–0.79)	0.44	93%	40%
**miR-192**	>0.05	1.13	0.61 (0.47–0.76)	0.4	96%	30%
**SNORD33**	>0.05	1.67	0.68 (0.55–0.82)	0.56	43.3%	96.3%
**SNORD37**	<0.05	0.07	0.82 (0.72–0.93)	0.78	93.3%	63.3%

* The p-values are based on Mann-Whitney test.

### ncRNAs as biomarkers in tissue samples of NSCLC patients

miR-223, miR-212, miR-192, SNORD33 and SNORD37 did not alter in tissue samples of cancer patients compared to non-cancers. Expression of miR-3074 significantly increased in tissue samples of cancer patients compared to non-cancers. miR-3074 overexpression resulted in 53% (95% CI, 0.63–1.00) sensitivity and 86% (95% CI, 0.86–1.00) specificity in the diagnosis of NSCLC (p<0.05) (Table 4). The prevalence of miR-223, miR-212, miR-192, miR-3074, SNORD33 and SNORD37 expression in tissue samples was not associated with patient age, gender, histological tumor type and stage (p>0.05).

**Table 4.** Evaluated ncRNAs in NSCLC patients and cancer-free controls in tissue samples

**ncRNA**	p-value*	**Fold change (cancer/control)**	**AUC (95% CI)**	**Cutoffs**	**Sensitivity**	**Specificity**
**miR-223**	>0.05	0.92	0.65 (0.50–0.79)	0.51	70%	56.7%
**miR-212**	>0.05	0.65	0.62 (0.47–0.77)	0.55	46.7%	93.3%
**miR-3074**	<0.05	3.6	0.73 (0.58–0.84)	0.71	86%	53.3%
**miR-192**	>0.05	0.79	0.47 (0.32–0.63)	0.53	73.3%	46.7%
**SNORD33**	>0.05	1.31	0.68 (0.53–0.83)	0.48	66.7%	86.7%
**SNORD37**	>0.05	1.07	0.55 (0.4–0.7)	0.5	80%	40%

* The p-values are based on Mann-Whitney test.

### Genetic changes in NSCLC patients and cancer-free individuals

**Figure 1.**
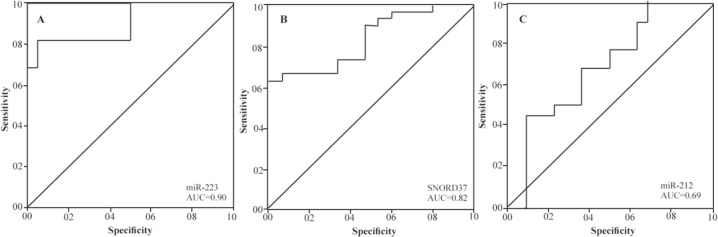
Receiver-operator characteristic (ROC) curve analysis of expression levels of the two miRNAs in sputum of 17 patients diagnosed with NSCLC and 17 healthy individuals. The area under the ROC curve (AUC) for each biomarker conveys its accuracy in distinguishing cancer-free subjects from cancer patients in terms of sensitivity and specificity. Significant genes produce (A, miR-223) 0.90 (95% CI, 0.81–0.99), (B, SNORD37) 0.82 (95% CI, 0.72–0.93) and (C, miR-212) 0.69 (95% CI, 0.53–0.85) AUC values.

The best AUC in our research belonged to miR-223 in sputum samples. Sensitivity and specificity of three significant biomarkers (miR-223, miR-212 and SNORD37) as a panel were not distinguishable from miR-223 alone in the diagnosis of NSCLC, sensitivity 82% (95% CI, 0.63–1.00) and specificity 95% (95% CI, 0.86–1.00) and AUROC at application of combined miR-223, miR-212 and SNORD37 in comparison to solitary miR-223 was not significant. Figure 1 shows ROC curve with corresponding AUROC for miR-223, miR-212 and SNORD37 expressions in sputum from cancer patients versus non-cancers.

## Discussion

Early diagnosis of NSCLC could change the disease outcome; actually, the survival rate will increase significantly. Many imaging and cytology-based strategies have been employed to augment early detection; however, because of low sensitivity or supererogatory cost, none has yet been highly efficient. Our current study clearly shows for the first time that alteration of miR-223 expression in sputum would provide a useful biomarker for noninvasive diagnosis of NSCLC.

Elevated miR-223 expression occurs in sputum of NSCLC patients with equal frequency among all histologic types of lung tumor, suggesting that the genetic changes are not specific to histologic type. Detection of the abnormality may be useful in determining different types of NSCLC that is really important since lung adenocarcinomas, which originate from the smaller peripheral airways, are difficult to be detected by bronchoscopy or sputum cytology and have become more prevalent than other types of lung cancer.

Ever since the first miRNA was discovered in C. elegans and was found to have an essential role in the worm development ^27,28^, it is a widely accepted concept that miRNAs are remarkable regulatory factors in development, apoptosis, and disease generation and progression ^29^. miRNAs participate in keeping the balance of genes regulating pathways that determine the cells’ fate. Deregulation of miRNAs incredibly withers this balance, thereby contributing to oncogenesis from initiation to metastasis.

Since there is no single validated molecular biomarker for early lung cancer detection and spectrum of biomarkers is needed for early diagnosis, a panel of biomarkers including miR-223, miR-212, miR-3074, miR-192, SNORD33 and SNORD37 were the candidates in our study.

miR-3074 is a less investigated member of miR-23b cluster that its dysregulation has been reported in various cancers ^30–34^. In our research, just expression of miR-3074 was significantly different in tissue samples of NSCLC patients compared with control group. However, in sputum samples, 3 biomarkers miR-223, miR-212 and SNORD37 significantly altered between cancer and control group.

miR-223 has been found to affect the cell cycle by regulating E2F1 ^35^, migration and invasion in cancer cells by targeting EPB41L3 ^36^, proliferation and tumor growth of cells by targeting IGF1R and downstream Akt/mTOR/p70S6K signaling pathway ^37,38^. miR-223 could act as a signal in the crosstalk between tumor and immune cells in the tumor microenvironment which leads to increased invasiveness in the cancer cells ^39^ or mediating immune evasion mechanisms ^40^. miR-223 affects different target genes at multiple cancers like Artemin (oesophageal carcinoma) ^41^, C/EBPβ (leukaemia) ^42^, E2F1 (leukaemia) ^35^, EPB41L3 (gastric cancer) ^36^, Fbxw7/Cdc4 (leukaemia, gastric cancer, oesophageal squamous cell carcinoma) ^43,44^, FOXO1 (colorectal cancer cells) ^45^, HSP90B1 (osteosarcoma) ^46^, IGF1R (HeLa, leukemia and hepatoma cells) ^37,47^, SEPT6 (prostate cancer) ^48^, LMO2 (Leukaemia/lymphoma) ^42^ and NFI-A (Leukaemia/lymphoma) ^49^. Because of important roles of miR-223, this biomarker was selected for investigation in NSCLC patients. In our research, miR-223 had the highest AUC between candidate biomarkers and significantly increased in patient group, but AUC 0.9 was not enough to be applied individually for diagnosis of NSCLC.

miR-212 was found to be dysregulated in many cancers: oral squamous cell carcinoma ^50^, colorectal carcinoma ^51^, gastric cancer ^52^, NSCLC ^53^ and head and neck squamous cell carcinoma ^54^ and recently, important biological functions in lung cancer cells for mir-212 has been proved ^17^. It had been reported that miR-212 was involved in cell cycle ^17^, DNA methylation ^52^ cell apoptosis ^53^, and signaling pathways ^55,56^. miR-212 significantly decreased in sputum of our lung cancer patients but sensitivity and specificity in the diagnosis of NSCLC were not sufficient for clinical trials; in addition, miR-212 did not improve compound sensitivity and specificity.

Several investigations suggest that snoRNAs exhibit differential expression in lung tumor and can affect cell transformation, tumorigenesis, and metastasis of NSCLC. SNORD33 and SNORD37 are located on chromosome 19q13.3 and 19p13.3, respectively that contain potential oncogenes involved in malignancies, including lung cancer ^20,57,58^. This study for the first time showed that SNORD37 significantly decreased in sputum samples of NSCLC patients and produced AUC=0.82 in distinguishing patient group from normal individuals. However, SNORD37 did not increase final sensitivity and specificity as a panel with miR-223 and miR-212.

Another characteristic of miRNAs unlike mRNAs is prominent stability in different kinds of biological specimens like urine, serum, plasma, saliva, sputum, formalin-fixed, paraffin-embedded clinical tissues and fresh snap-frozen materials ^59–64^. This prominent stability is due to their resistance to endogenous and exogenous RNase activity, extreme temperatures and pH, long storage in frozen conditions, and repeated freezethaw cycles ^61,63,65^. These features introduce miRNA as a great target for different aspects of biological and medical investigations. Although miRNA has recently emerged as a powerful molecular biomarker for detection of diseases like cancers, its potential as a sputum-based biomarker has not been fully explored.

Sputum has the benefits as a potential surrogate substance for molecular genetic diagnosis of lung cancer, because its non-invasive procurement would allow the comprehensive analysis of tumors without the requirement of invasive procedures, such as biopsy or surgery, and the fact that it contains clinically worthy lung and lower respiratory tract bronchial epithelial cells adds to its benefit. Furthermore, sputum has low cost and sample management, including sample collection and processing is simple ^66^.

## Conclusion

Although assessment of miR-223 expression in sputum seems to be hopeful in the noninvasive detection of lung cancer, 82% sensitivity and 95% specificity are not efficient for routine clinical application. In this study, although the sputum and tissue levels of some biomarkers in NSCLC patients were analyzed at different stages, the number of patients was small and the number of biomarkers tested was limited. In the future investigation, more samples especially early-stage samples should be accessed to evaluate the role of sputum miRNAs associated with NSCLC. The outcome might indicate the need to develop a strategy for simultaneous evaluation of a panel of tumor-specific miRNA biomarkers in sputum in order to attain an extremely sensitive and specific diagnostic test for lung cancer.
